# miR-876-3p is a tumor suppressor on 9p21 that is inactivated in melanoma and targets ERK

**DOI:** 10.1186/s12967-024-05527-7

**Published:** 2024-08-13

**Authors:** Vladimir Bezrookove, Imran Khan, Anukana Bhattacharjee, Juifang Fan, Robyn Jones, Anima Sharma, Mehdi Nosrati, Pierre-Yves Desprez, Nathan Salomonis, Yihui Shi, Altaf Dar, Mohammed Kashani-Sabet

**Affiliations:** 1 California Pacific Medical Center (CPMC) Research Institute, 475 Brannan St., Suite 130, San Francisco, CA 94107 USA; 2 Center for Melanoma Research and Treatment, CPMC, San Francisco, CA USA; 3 Division of Biomedical Informatics, Cincinnati Children’s Hospital Medical Center, Cincinnati, OH USA

**Keywords:** Melanoma, microRNA-876, Tumor suppressor, Mitogen activated protein kinase

## Abstract

**Background:**

While melanomas commonly harbor losses of 9p21, on which *CDKN2A* resides, the presence of additional tumor suppressor elements at this locus is incompletely characterized. Here we assess the expression levels and functional role of microRNA-876-3p (miR-876), whose gene also maps to 9p21.

**Methods:**

Expression of miR-876 was assessed in human tissues and cell lines using quantitative miRNA reverse transcriptase polymerase chain reaction (qRT-PCR). *MIR876* copy number was determined in The Cancer Genome Atlas (TCGA) melanoma cohort. The consequences of regulation of miR-876 expression were assessed on melanoma cell colony formation, migration, invasion, apoptosis, cell cycle progression, and drug sensitivity in culture, and on in vivo tumor growth in a xenograft model. Genome-wide transcriptomic changes induced by miR-876 overexpression were determined using RNA sequencing (RNA-Seq).

**Results:**

miR-876 expression was significantly decreased in primary melanoma samples when compared with nevi, and in human melanoma cell lines when compared with human melanocytes. Analysis of the TCGA cohort revealed deletions in *MIR876* in > 50% of melanomas. miR-876 overexpression resulted in decreased melanoma cell colony formation, migration, and invasion, which was accompanied by cell cycle arrest and increased apoptosis. Intra-tumoral injections of miR-876 significantly suppressed melanoma growth in vivo. RNA-Seq analysis of miR-876-treated tumors revealed downregulation of several growth-promoting genes, along with upregulation of tumor suppressor genes, which was confirmed by qRT-PCR analysis. Computational analyses identified *MAPK1* (or *ERK2*) as a possible target of miR-876 action. Overexpression of miR-876 significantly suppressed luciferase expression driven by the *MAPK1*/*ERK2* 3’ UTR, and resulted in decreased ERK protein expression in melanoma cells. *MAPK1*/*ERK2* cDNA overexpression rescued the effects of miR-876 on melanoma colony formation. miR-876 overexpression sensitized melanoma cells to treatment with the BRAF inhibitor vemurafenib.

**Conclusions:**

These studies identify miR-876 as a distinct tumor suppressor on 9p21 that is inactivated in melanoma and suggest miR-876 loss as an additional mechanism to activate ERK and the mitogen activated protein kinase (MAPK) pathway in melanoma. In addition, they suggest the therapeutic potential of combining miR-876 overexpression with BRAF inhibition as a rational therapeutic strategy for melanoma.

**Supplementary Information:**

The online version contains supplementary material available at 10.1186/s12967-024-05527-7.

## Background

Melanoma is the fifth most common malignancy in the United States, with an estimated 100,640 cases and 8290 deaths in 2024 [1]. Melanomas are characterized by a high degree of molecular aberrations, including cytogenetic alterations as well as a high mutational burden [2]. Specifically, a majority of melanomas have been shown to harbor losses on the short arm of chromosome 9, principally involving the 9p21 locus [3, 4]. Losses of the *CDKN2A* gene, which resides on 9p21, have been shown in a substantial proportion of both familial and sporadic melanomas [5–7]. In addition, loss of the methylthiadenosine phosphorylase (*MTAP*) gene, which also maps to 9p21, has been shown in melanoma [8, 9]. However, whether this locus contains other genes with tumor suppressor properties that are inactivated in melanoma remains poorly characterized. In addition, a high proportion of melanomas show activation of the mitogen activated protein kinase (MAPK) pathway, most commonly with mutations involving the *BRAF*, *NRAS*, and *NF1* genes [2], ultimately resulting in activation of the ERK protein, which represents a critical downstream effector of the MAPK pathway that drives tumor cell invasion, proliferation, and metastasis [10]. However, a subset of melanomas lacks activating mutations in these MAPK pathway genes, suggesting that alternative mechanisms of MAPK pathway activation may be present.

MicroRNAs (miRNAs) are single strand RNAs that regulate expression of target genes. miRNAs have emerged as important epigenetic regulators of many cellular processes contributing to tumorigenesis, including tumor cell proliferation, apoptosis, and invasion. miRNAs have been shown to be either upregulated or downregulated in various malignancies and shown to play important roles in tumor progression. Several miRNAs have been shown to be differentially expressed in melanoma and/or to play important roles in melanoma progression [11, 12]. In this study, we explored the role of miRNA-876-3p in melanoma progression, given that the gene encoding miR-876 resides on 9p21, and given prior studies demonstrating a tumor suppressor role for miR-876-3p (hereafter miR-876) in various solid tumors [13–15]. We demonstrate a functional tumor suppressor role for miR-876 in melanoma and identify ERK as a target of miR-876 action.

## Methods

### Cell culture

A panel of melanoma cell lines was utilized to assess the expression of miR-876, including C8161.9 (obtained from Dr. D. Welch, UAB, USA), A375 (ATCC, Manassas, VA, USA), LOX (a gift from Dr. Oystein Fodstad, University of Oslo, Norway), 1205-Lu (Coriell Institute, Camden, NJ, USA), and Ma-Mel-12, a short-term melanoma culture (a gift from Dr. Dirk Schadendorf, University of Essen, Germany), as previously described [16]. Human epidermal melanocytes (HEM), used as a control, were obtained from Zen-Bio (Durham, NC, USA), and grown in Mel-2 melanocyte medium. Further characterization of the role of miR-876 was performed in C8161.9 and A375 cells. C8161.9 cells were grown in DMEM/F12 with 5% fetal bovine serum (FBS) (Invitrogen Life Technologies, Carlsbad, CA, USA), and A375 cells were grown in RPMI-1640 with 5% FBS. Cell culture media was supplemented with 1X penicillin/streptomycin (Thermo Fisher Scientific, South San Francisco, CA, USA) and cells were grown at 37 ºC and 5% CO_2_. All cell lines were routinely tested for mycoplasma contamination using the MycoFluor Mycoplasma Detection Kit (Thermo Fisher Scientific, South San Francisco, CA, USA) following the manufacturer’s instructions.

### Plasmids and transfection

The pmir GLO-dual luciferase vector (Promega, Madison, WI, USA), pEZX-MR04-miR-876 and pEZX-MR04 control vectors (GeneCopoeia, Rockville, MD, USA) and the *MAPK1/ERK2* cDNA expression vector (Origene, Rockville, MD, USA) were purchased. Transient transfection was carried out by Lipofectamine-2000 (Invitrogen Life Technologies, Carlsbad, CA, USA) according to the manufacturer's protocol. Briefly, 50 nM of control miR (termed cont. miR) or miR-876 were used for transfection. miRNAs were mixed with lipofectamine in serum-free medium and the reaction mixture was added to the cells for 4 h, after which the media was aspirated and replaced. Stable transformants were generated by transfecting C8161.9 and A375 cells with pEZX-MR04 control and pEZX-MR04-876 vectors following selection with puromycin (1 µg/mL). Stable transformants were sorted based on GFP expression using FACS Aria II (BD Biosciences). Transfection of the *MAPK1/ERK2* cDNA into C8161.9 and A375 cells was performed as previously described [17].

### miRNA extraction and quantitative reverse transcriptase polymerase chain reaction (qRT-PCR)

Samples from primary melanomas (N = 55) and nevi (N = 48) were collected under the auspices of a protocol approved by the Sutter Health Institutional Review Board. The histologic nevus subtypes included in the cohort are presented in Supplemental Table 1. miRNAs from melanoma cell lines and human tumor tissues were extracted by using the mirVana miRNA extraction kit (Thermo Fisher Scientific, South San Francisco, CA) following the manufacturer’s instructions. Quantitation of miR-876 expression in tissues and cell lines was performed by qRT-PCR as previously described [18]. pre-miR-miRNA precursor molecule-negative control (referred to as control miR) and pre-miR-miRNA-876 precursor (referred to as miR-876) and their corresponding miRNA Taqman probes were purchased from ThermoFisher Scientific (South San Francisco, CA, USA). Mature miRNAs were assayed using the TaqMan MicroRNA Assays in accordance with the manufacturer's instructions (Thermo Fisher Scientific, South San Francisco, CA, USA). All RT reactions, including no-template controls and RT minus controls, were run in a 7500 Fast Real Time PCR System (Thermo Fisher Scientific, South San Francisco, CA, USA). RNA concentrations were determined with a NanoDrop (Thermo Fisher Scientific, South San Francisco, CA, USA). Samples were normalized to RNU44 (Thermo Fisher Scientific, South San Francisco, CA, USA). For the validation of RNA-Seq results, RNA was extracted from three tumors per treatment group or following transfection of miR-876 or control miR into C8161.9 cells in culture, and cDNA synthesis and qRT-PCR were performed as described previously [16]. Quantitative PCR was performed using Taqman probes (Thermo Fisher Scientific) for *TNFRSF4* (Hs00937195_g1), *DDR1* (Hs01058430_m1), *CA9* (Hs00154208_m1), *CDC42EP5* (Hs01936746_s1), *VEGFB* (Hs00173634_m1), *BRCA2* (Hs00609073_m1), *BLM* (Hs01119891_m1). *GAPDH* (Hs02786624_g1), *HPRT1* (Hs02800695_m1), and *TBP* (Hs00427621_m1) were used as control genes. Gene expression levels were quantified using the 7500 Fast Real Time Sequence detection system software (Thermo Fisher Scientific, South San Francisco, CA, USA). Comparative real-time PCR was performed in triplicate, including no-template controls. Relative expression was calculated using the comparative Ct method. Experiments were repeated three times in triplicate for cell lines and twice for tissues.

### Luciferase expression assay

Assessment of luciferase expression was performed as previously described [18]. Briefly, the 3’ UTR region of the *MAPK1*/*ERK2* gene containing target site sequences complementary to the seed sequence of miR-876 was cloned in the pmir GLO-dual luciferase vector (Promega, Madison, WI, USA), and the resultant vector named ERK-UTR. For reporter assays, cells were transiently transfected with reporter plasmid with control miR or miR-876. Firefly luciferase activities were measured by using the Dual Luciferase Assay (Promega, Madison, WI, USA) 24 h after transfection and the results were normalized with Renilla luciferase. The experiments were performed in triplicate.

### Colony formation assay

The colony formation assay was performed as previously described [18]. Briefly, 500–700 cells were plated in each well of a 6-well plate and allowed to grow till visible colonies appeared. Colonies were stained with crystal violet and counted. The experiments were performed in triplicate.

### Western blotting

Cell lysates were prepared in RIPA buffer containing 1 × Halt protease inhibitor cocktail and 1 × Halt phosphatase inhibitor cocktail (Pierce, Rockford, IL, USA) centrifuged at 3500 rpm for 10 min at 4 °C. Proteins (25–50 µg) from each sample were subjected to SDS/polyacrylamide gel electrophoresis (PAGE) and transferred onto a nitrocellulose membrane. Western analysis of ERK expression was performed as previously described [17] using specific antibodies against ERK1/2 (#4695, from Cell Signaling Technology, Danvers, MA) and GAPDH (#sc-365062, from Santa Cruz Biotechnology, Santa Cruz, CA, USA).

### Cell cycle and Annexin V assays

Cell cycle and Annexin V assays were performed by using the Muse cell cycle kit and Muse Annexin V apoptosis kit, respectively (EMD Millipore, Hayward, CA, USA) as described [18].

### Migration and invasion assays

Melanoma cells (1.5 × 10^5^) were seeded in the upper wells of BD BioCoat Chambers (BD Biosciences, Bedford MA), with or without 15 µL of Matrigel at 6 mg/mL in RPMI without serum. The lower wells contained the same medium with 20% serum. The cells that invaded or migrated 24 h later were fixed, stained and counted.

### Cell counting kit-8 (CCK-8) cell proliferation assay

A375 cells stably expressing control miR or miR-876 were seeded (1000 cells/well in 100 μL medium) in a 96-well cell culture plate (Corning Inc., Corning, NY, USA) on Day − 1 and incubated at 37 °C in a humidified incubator with 5% CO_2_ for 24 h. On Day 0, the culture medium was replaced with medium containing various concentrations of vemurafenib, starting at 10 µM and serially diluted by a factor of three. Cells were incubated for another 72 h under the same conditions as described above. On Day 3, 10 μL of the CCK-8 reagent (Dojindo Molecular Technologies, Rockville, MD) was added into each well, and the OD at 450 nm was measured using a multimode microplate reader (Varioskan, Thermofisher Scientific, Chicago, IL, USA) after incubation for 2 h at 37 °C.

### Animal studies

Eight-week-old *nu*/*nu* mice were purchased from Jackson Laboratories, Sacramento, CA, USA. In vivo studies were carried out in accordance with the National Institutes of Health guidelines, Health Research Extension Act of 1985 and the Public Health Service Policy on Humane Care and Use of Laboratory Animals (Policy), Office of Laboratory Animal Welfare assurance, and an approved Institutional Animal Care and Use Committee (IACUC) protocol. C8161.9 cells (1 × 10^6^) were injected subcutaneously in a total volume of 100 µL in the mouse flank. Once tumors were palpable (with average tumor volumes ≥ 70 mm^3^), mice were randomized and divided into the following treatment groups: control miR (n = 8) and miR-876 (n = 8). The miR was injected intratumorally twice weekly for the duration of the study, as previously described [19]. The animals were randomly assigned to the treatment groups, and the investigator performing tumor measurements was blinded to the identity of the treatment groups. No samples were excluded from the analysis. Tumors were measured by caliper and volumes were calculated as a product of (length × width × width)/2. Mice were sacrificed and tumors collected and processed for RNA extraction.

### RNA sequencing (RNA-Seq)

RNA extraction and sequencing were performed as previously described [20]. Briefly, total RNA was extracted from tumor tissues using the RNeasy tissue kit (Qiagen, Redwood City, CA, USA). RNA-Seq was performed from ~ 500 ng of total RNA processed using TruSeq polyA selection, at a target depth of 40 million paired-end, stranded reads on an Illumina 2500. The RNA-Seq data was aligned to the human reference genome (hg19) using the software STAR, followed by gene quantification in the software AltAnalyze to obtain gene-level RPKM values. Differential expression (fold > 1.2, empirical Bayes moderated t-test p < 0.05) was determined using AltAnalyze version 2.1.3 using the Ensembl 72 human database. Embedded gene-set enrichment analyses were performed using GO-Elite with default options. Hierarchical clustering was performed in AltAnalyze using HOPACH clustering for rows and weighted cosine clustering for genes. The analyses presented focused on a heat map representing the top 684 up- and down-regulated genes (empirical Bayes moderated t-test, p < 0.05; fold change = 1.2).

### Next-generation sequencing (NGS)

#### Tumor DNA extraction

Genomic DNA was extracted from the panel of melanoma cell lines using the QIAamp DNA FFPE Tissue kit (Qiagen, Redwood City, CA, USA), and its quantity and integrity determined using a TapeStation (Agilent Technologies, Santa Clara, CA, USA).

#### DNA sequencing

MiSeq 2 × 151 base paired-end sequencing was performed to detect single-nucleotide variant (SNV) and insertion/deletion (indel) variants at 1% allelic frequency or higher in target regions with sufficient read coverage. We used the 56G Oncology Panel V2 from Swift Biosciences (Ann Arbor, MI, USA). Per sample, we only considered a mean coverage of at least 500 for DNA sequencing, and SNV and indel variants at 1% allelic frequency or higher in target regions with sufficient read coverage (at least 100 ×). The 56 gene targets covered by the cancer panel were previously described [21] but did include *BRAF* and* CDKN2A.*

#### Data analysis

Data obtained using the 56G Oncology Panel V2 was analyzed using Genialis Expressions (Accel-Amplicon analysis workflow, Genialis Inc., Boston, MA, USA). In brief, quality trimmed (Trimmomatic v.0.36) sequencing data was aligned to the human genome (GRCh37 assembly) using BWA MEM (v. 0.7.17-r1188). The aligned data was further processed by trimming primer sequences (Primerclip, Swift biosciences) and GATK (v.3.6) tools (IndelRealigner and BaseRecalibrator) to prepare analysis ready BAM file. SNP/INDELs were called using LoFreq (v.2.1.3.1) and annotated using snpEff (v.4.3k).

### Fluorescence in situ hybridization (FISH)

FISH for analysis of copy number was performed as previously described [22, 23] using bacterial artificial chromosome clones (BAC) for *CDKN2A* (RP11-149I2) and a clone mapping to the centromeric region of chr. 9p11.2 (RP11-69O9) based on the December 2013 freeze of UCSC Genome Browser (http://genome.ucsc.edu). All BAC clones were obtained from the Children's Hospital Oakland Research Institute. The quality and mapping of all probes were verified by hybridization to normal metaphase spreads in combination with commercially available centromeric probe (Empire Genomic, New York, USA). Z-stacked images were acquired using a Zeiss Axio Image Z2 microscope controlled by AxioVision software (Zeiss, Jena, Germany). At least 40 nuclei from each case were evaluated, and the signals were interpreted according to guidelines described previously [24].

### Statistical analysis

All quantified data represent an average of at least triplicate samples or as indicated. Statistical significance was determined using the Student’s t-test or Mann–Whitney U test, and P values < 0.05 were considered significant. Data are represented by the mean with error bars representing standard deviation. In the in vivo anti-tumor study, sample sizes were determined prospectively, using a type I error rate of 0.05 and power of 0.8 to detect differences in mean tumor volume of at least 30%.

## Results

**Fig. 1:**
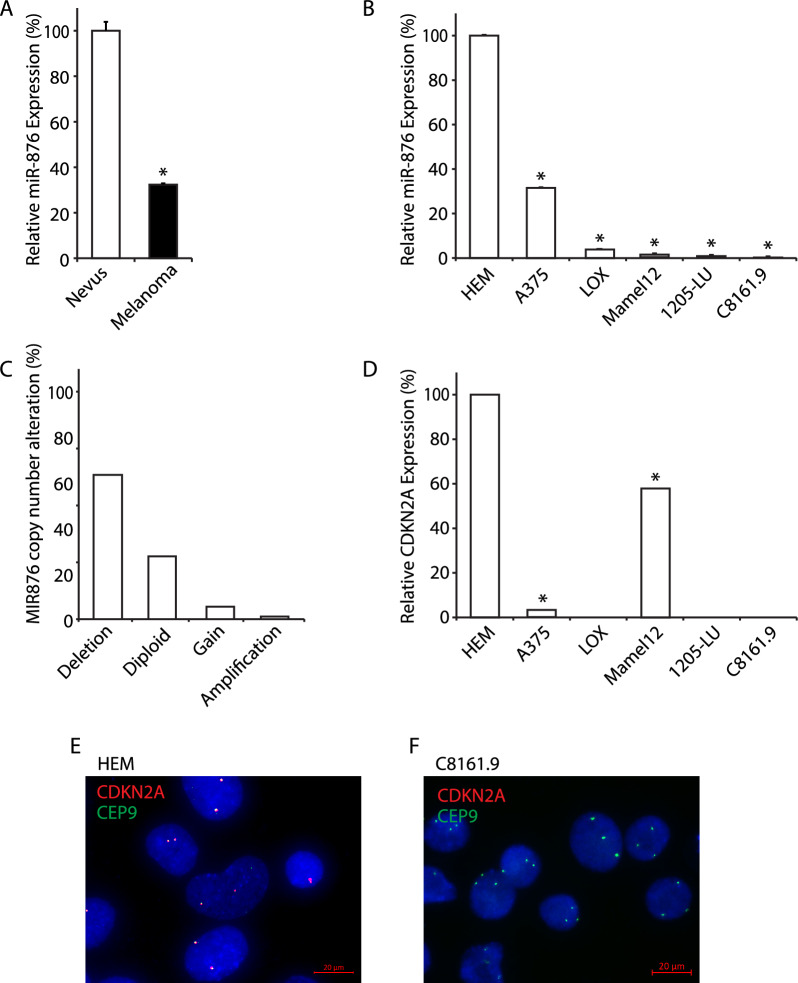
miR-876 and *CDKN2A* expression and copy number in human melanoma samples and cell lines. **A** Relative expression by qRT-PCR of miR-876 in melanoma samples (n = 55) compared to nevi (n = 48). **B** Relative expression by qRT-PCR of miR-876 in a panel of melanoma cell lines normalized to a human melanocyte (HEM) cell line. **C** Analysis of miR-876 copy number variation in the TCGA melanoma cohort. **D** Relative expression by qRT-PCR of *CDKN2A* in a panel of human melanoma cell lines normalized to a human melanocyte (HEM) cell line. **E**, **F** Representative FISH images detecting the *CDKN2A* locus in red and CEP9 in green in HEM (panel **E**) and C8161.9 cells (panel **F**). Data are shown as the mean ± SEM. *P < 0.05 denotes statistically significant differences compared with control. Scale bar, 20 µm

Initially, we aimed to determine the expression levels of miR-876 in human melanoma tissues and cell lines when compared to benign nevi and normal human melanocytes, respectively. We performed qRT-PCR analysis of miR-876 expression in a tissue cohort of 55 melanomas and 48 nevi and observed ~ 70% downregulation of miR-876 expression in primary melanomas when compared to melanocytic nevi (Fig. 1A). We also assessed miR-876 expression in a panel of human melanoma cell lines when compared to the HEM human melanocyte cell line. miR-876 expression was significantly downregulated in each melanoma cell line examined, with profound levels of downregulation (> 80%) in 4 out of the 5 cell lines examined (Fig. 1B). Subsequently, we assessed *MIR876* copy number levels in the TCGA melanoma cohort and observed deletions in ~ 60% of cases (Fig. 1C). Taken together, these results demonstrate *MIR876* copy number loss as well as suppressed expression levels in a substantial cohort of melanoma specimens and cell lines, supporting a tumor suppressor role.

In addition, we determined *CDKN2A* expression levels, copy number, and mutational status in the same panel of cell lines examined using qRT-PCR, FISH, and NGS analysis, respectively. qRT-PCR analysis showed significant downregulation of *CDKN2A* expression in all melanoma cell lines examined when compared with the HEM melanocyte line (Fig. 1D). FISH analysis indicated euploidy for *CDKN2A* in HEM cells (Fig. 1E), whereas C8161.9 cells harbored copy number loss for *CDKN2A* (Fig. 1F). Separately, NGS analysis identified two distinct point mutations in *CDKN2A* (Glu61* and Glu69*) in the A375 cell line, consistent with prior reports [25]. Taken together, these results indicate that expression of miR-876 is downregulated in a substantial proportion of melanoma cell lines, along with reduced expression of *CDKN2A* in the same cell lines.

**Fig. 2:**
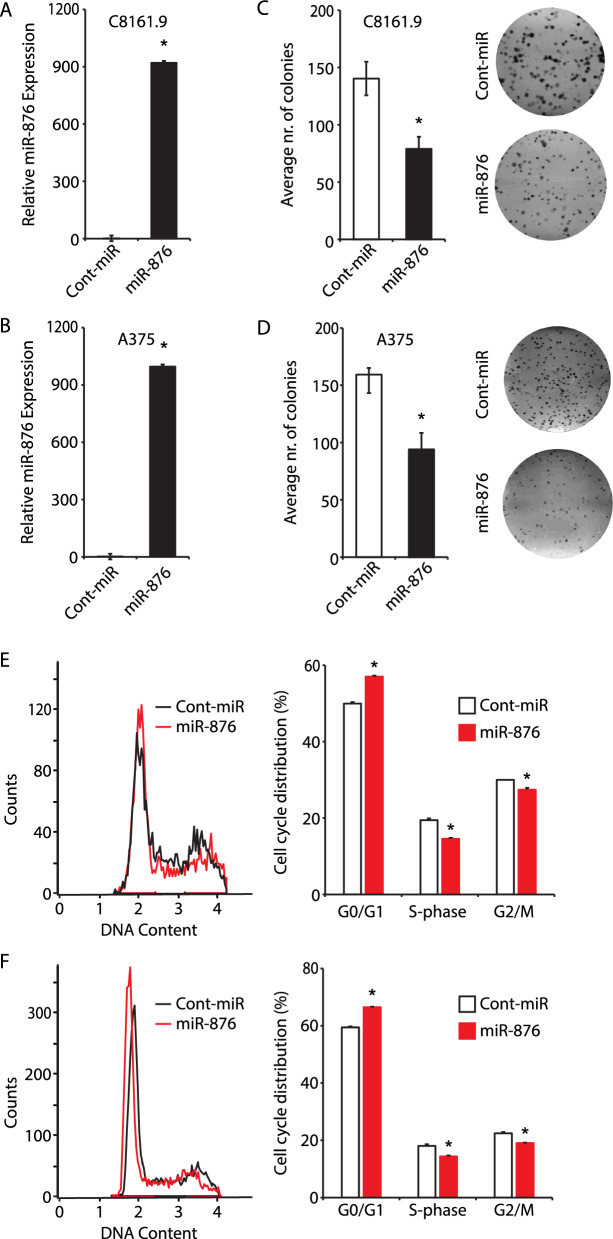
Effects of miR-876 overexpression on melanoma colony formation and cell cycle progression. **A**, **B** Relative expression by qRT-PCR of miR-876 (panel **A**) in C8161.9 and A375 (panel **B**) stable transformants. **C, D** Bar graphs showing the average number of colonies in C8161.9 (panel **C**) and A375 (panel **D**) cells stably expressing miR-876 compared to a control-miR (termed cont. miR) sequence, along with representative bright field images of C8161.9 and A375 colonies in culture stably expressing miR-876 vs control-miR. Data are shown as the mean ± SEM. *P < 0.05 denotes statistically significant differences compared with control. **E**, **F** Cell cycle profile with accompanied analysis of distribution of C8161.9 (panel **E**) and A375 melanoma (panel **F**) cells stably expressing either miR-876 or control-miR in different phases of the cell cycle

**Fig. 3:**
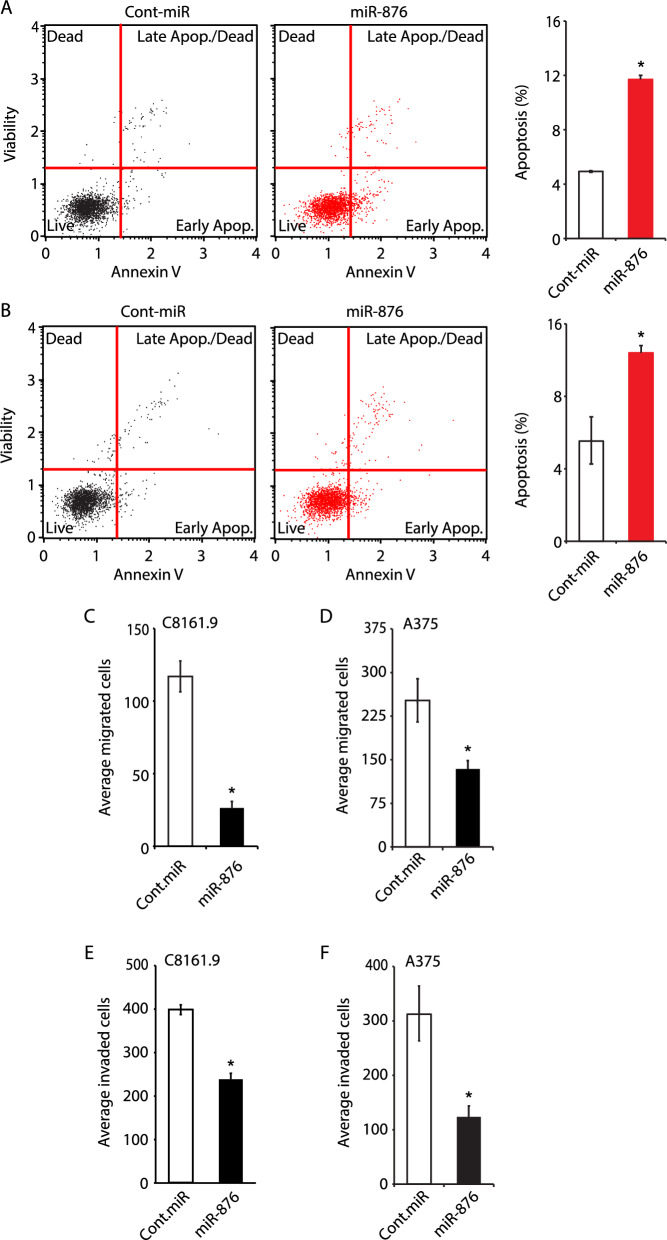
Effects of miR-876 overexpression on apoptosis, migration and invasion in melanoma cells. **A, B** Analysis of apoptotic rates with representative dot plots and bar graphs indicating percentage of total apoptotic C8161.9 (panel **A**) and A375 cells (panel **B**) stably expressing miR-876 compared to control-miR. **C, D** Migratory capacity of C8161.9 (panel **C**) and A375 cells (panel **D**) stably expressing miR-876 compared to control-miR. **E, F** Invasive capacity of C8161.9 (panel **E**) and A375 cells (panel **F**) stably expressing miR-876 compared to control-miR. Data are shown as the mean ± SEM. *P < 0.05 denotes statistically significant differences compared with control

We next aimed to determine whether miR-876 functionally acts as a tumor suppressor. Stable overexpression of miR-876 in C8161.9 (Fig. 2A) and A375 (Fig. 2B) cells resulted in significant suppression of the colony formation capacity of melanoma cells (Fig. 2C, D). miR-876 overexpression was accompanied by significant changes in the cell cycle distribution of C8161.9 (Fig. 2E) and A375 cells (Fig. 2F), including G1 arrest and reduced S phase population. In addition, miR-876 overexpression resulted in significant induction of apoptosis in C8161.9 (Fig. 3A) and A375 cells (Fig. 3B). Lastly, overexpression of miR-876 suppressed the migratory (Fig. 3C, D) and invasive (Fig. 3E, F) capacity of melanoma cells. These studies indicate that miR-876 regulates human melanoma cell growth, migration, invasion, and apoptosis, consistent with a functional tumor suppressor role.

**Fig. 4:**
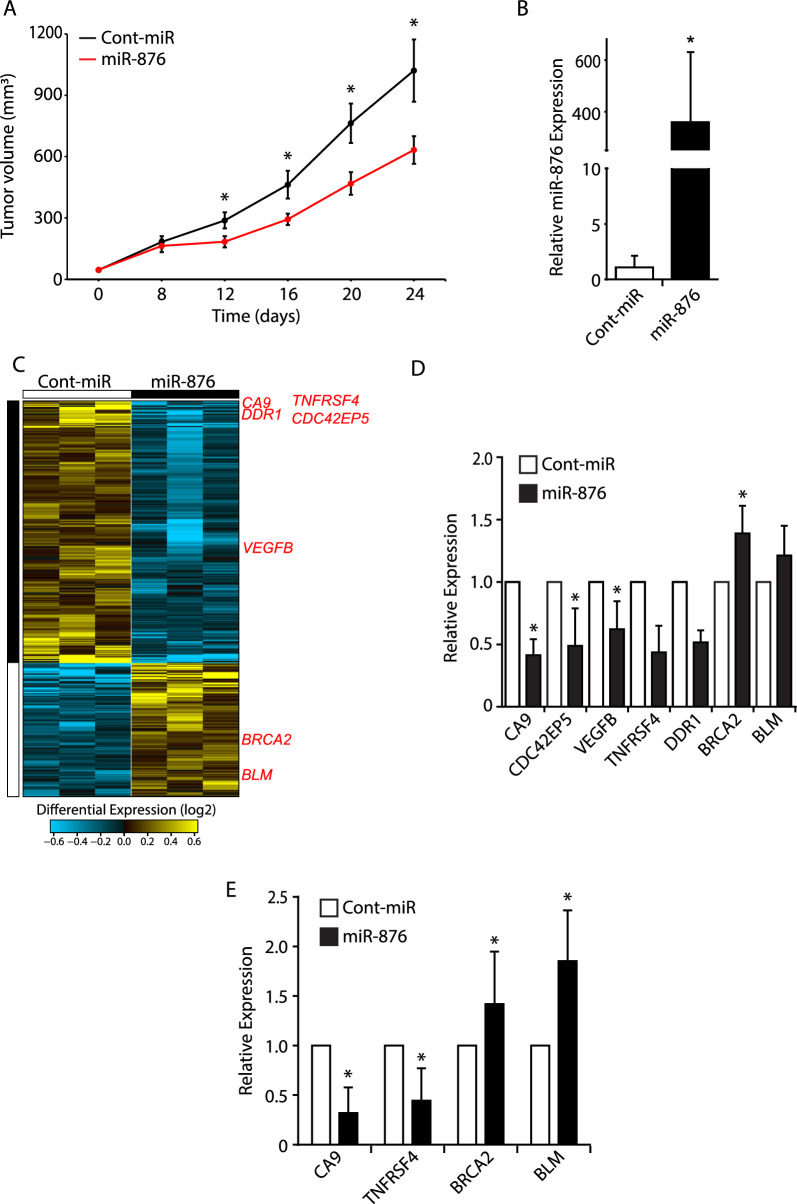
The effects of miR-876 overexpression on C8161.9 melanoma cells in vivo. **A** Tumor volume of C8161.9 cells subcutaneously injected in nude mice following intratumoral treatment with miR-876 or control-miR twice weekly for 24 days. **B** Relative miR-876 expression by qRT-PCR in C8161.9 in vivo tumors treated with miR-876 or control (N = 4 per group). **C** RNA-Seq followed by supervised hierarchical analysis of C8161.9 tumors treated in vivo with either miR-876 or control-miR (N = 3 per group). **D** Relative expression by qRT-PCR of differentially expressed genes in C8161.9 tumors (N = 3 per group). **E** Relative expression by qRT-PCR of differentially expressed genes in C8161.9 cells grown in culture following transfection with miR-876 or control miR. Data are shown as the mean ± SEM. *P < 0.05 denotes statistically significant differences compared with control

Subsequently, we aimed to assess the consequences of restoration of miR-876 expression in melanoma cells in vivo. C8161.9 cells were subcutaneously injected into nude mice, and the resultant tumors received intratumoral injections of miR-876 or control miR twice weekly for 24 days. There was a significant reduction in tumor volume of miR-876-treated tumors versus control (P < 0.05, Fig. 4A), accompanied by significantly increased miR-876 levels in treated tumors (Fig. 4B). These studies demonstrate a functional tumor suppressor role for miR-876 in melanoma in vivo. In order to understand the genome-wide transcriptomic changes induced by miR-876 treatment, we performed bulk RNA-Seq on miR-876- and control miR-treated C8161.9 tumors. Supervised analysis identified numerous differentially expressed genes, including 2,469 downregulated and 702 overexpressed genes (Fig. 4C, Supplemental Table 2). Gene ontology analysis identified tumor suppressor genes, homologous recombination, and ATM/ATR signaling as significantly upregulated (Supplemental Table 3). Specifically, the expression of *CA9*, *CDC42EP5*, *VEGFB, TNFRSF4* and *DDR1* was downregulated following miR-876 treatment, whereas expression of *BRCA2* and *BLM* was upregulated. The differential expression of several of these genes was confirmed using qRT-PCR analysis in treated tumors (Fig. 4D) and in culture following miR-876 overexpression (Fig. 4E).

**Fig. 5:**
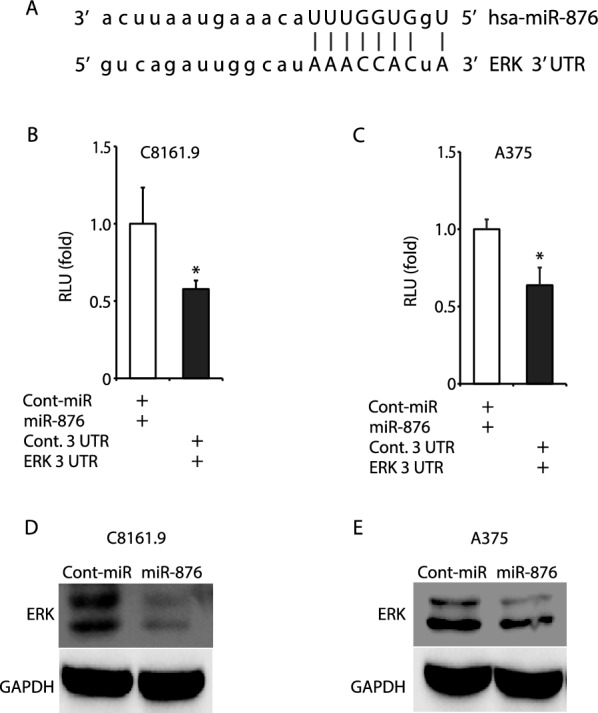
ERK as a potential target of miR-876. **A** The putative binding sites of miR-876 in the 3′UTR of *MAPK1*/*ERK2*. **B, C** Luciferase reporter assays verifying the regulation of miR-876 in the 3’UTR of *MAPK1*/*ERK2* in C8161.9 (panel **B**) and A375 (panel **C**) cells transiently co-transfected with miR-876 (or control-miR) and a plasmid encoding the luciferase reporter gene and *MAPK1*/*ERK2* 3’ UTR. **D, E** Western blot analysis of ERK expression in C8161.9 (panel **D**) and A375 (panel **E**) expressing miR-876 vs control-miR

Finally, we performed computational analyses of various databases (e.g., TargetScan and miRanda) to determine possible targets of miR-876, and identified *MAPK1*/*ERK2* as a putative target, given the complementarity of the seed sequence of miR-876 with the 3’ UTR of *ERK* (Fig. 5A). To determine whether miR-876 regulates *MAPK1*/*ERK2*, we cloned the *ERK* 3’ UTR into a plasmid encoding the luciferase reporter gene. Transient co-transfection of the *ERK*-3’ UTR construct along with miR-876 into C8161.9 (Fig. 5B) and A375 (Fig. 5C) human melanoma cells resulted in significant downregulation of luciferase expression when compared with a control vector. In addition, overexpression of miR-876 into C8161.9 (Fig. 5D) and A375 (Fig. 5E) cells resulted in substantial downregulation of ERK protein expression. These results identify ERK as a target of miR-876 action in human melanoma cells.

**Fig. 6:**
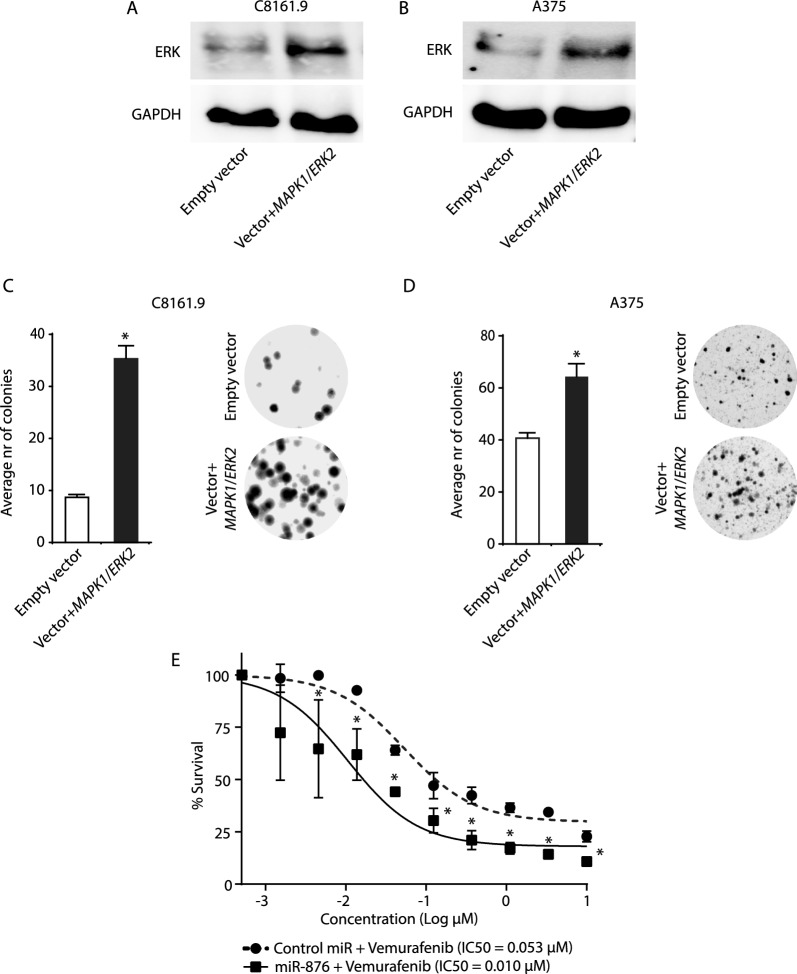
Effects of regulation of ERK by miR-876 on melanoma colony formation and drug sensitivity. **A, B** Western analysis of ERK expression in C8161.9 (panel **A**) and A375 (panel **B**) cells stably expressing miR-876 transfected with a plasmid encoding *MAPK1*/*ERK2* cDNA or empty vector. **C, D** Bar graphs showing the average number of colonies in C8161.9 (panel **C**) and A375 (panel **D**) cells stably expressing miR-876 transfected with a plasmid encoding *MAPK1*/*ERK2* cDNA or empty vector, with accompanying representative bright field images of C8161.9 and A375 colonies. **E** Viability of A375 cells expressing miR-876 or control miR following treatment with various concentrations of vemurafenib. Data are shown as the mean ± SEM. *P < 0.05 denotes statistically significant differences compared with control

Subsequently, we performed rescue experiments by transfecting *MAPK1*/*ERK2* cDNA or an empty vector into melanoma cells stably expressing miR-876. *MAPK1*/*ERK2* cDNA overexpression (Fig. 6A, B) resulted in significantly increased colony formation capacity of miR-876-expressing C8161.9 (Fig. 6C) and A375 (Fig. 6D) cells following *MAPK1*/*ERK2* cDNA overexpression. Finally, we assessed whether downregulation of ERK following miR-876 overexpression could alter sensitivity to BRAF inhibitor treatment. To this end, stable miR-876-expressing (and control miR-expressing) A375 cells (harboring a *BRAF* V600E mutation) were treated with various concentrations of the selective BRAF inhibitor vemurafenib. miR-876 overexpression resulted in significantly increased sensitization to vemurafenib administration (Fig. 6E), with a five-fold lower IC_50_ than that observed with control miR-expressing cells.

## Discussion

In this study, we examined the tumor suppressor role of miR-876 in human melanoma. At the molecular level, melanoma is characterized by activation of oncogenes and loss of tumor suppressor genes. To date, the identification of numerous genetic alterations in melanoma [2] has advanced our understanding of its etiology and pathogenesis. Analysis of the TCGA melanoma cohort described oncogenic activation of driver mutations such as *BRAF*, *NRAS*, and *CKIT*, whereas the tumor suppressor genes identified included *CDKN2A*, *NF1*, *PTEN*, and *TP53*. Our study adds to the knowledge of melanoma biology by adding miR-876 to the list of tumor suppressors inactivated in melanoma.

The identification of an additional tumor suppressor on 9p21 in melanoma is of particular interest and significance. This is the most commonly altered chromosomal locus in melanoma [26], and has been known to harbor at least two tumor suppressors, *CDKN2A* and *MTAP*. Our studies extend these findings by demonstrating the presence of another gene with tumor suppressor properties on 9p21. Importantly, miR-876 expression was profoundly downregulated in both melanoma tissues and cell lines, with evidence of downregulated *CDKN2A* expression also being present in the same panel of melanoma cell lines. Analysis of the TCGA melanoma cohort indicated evidence of *MIR876* copy number loss in a majority of melanoma samples. These observations suggested a tumor suppressor role for miR-876 in melanoma, which was supported by functional studies showing that miR-876 overexpression resulted in significant suppression of melanoma cell colony formation, migration, invasion, and cell cycle progression, along with activation of apoptosis. The tumor suppressor role of miR-876 was further confirmed in vivo in a xenograft melanoma model. To our knowledge, these studies are the first to describe miR-876 downregulation in melanoma, along with functional tumor suppressor activity. Our study thus adds miR-876 to the growing list of miRNAs that are inactivated in melanoma, with a putative or demonstrated tumor suppressor role [11, 12]. Previously, we showed that miR-876-3p acts as a tumor suppressor in cholangiocarcinoma [14], consistent with a similar role in other solid tumors [15]. Taken together, these studies assign a broad-based tumor suppressor role for miR-876-3p.

The identification of ERK as a target of miR-876 action is also of interest, given the dominant role played by MAPK pathway activation in melanoma. To date, most of the studies of MAPK pathway activation have focused on mutational activation of the pathway, including that of *BRAF*, *NRAS*, and *NF1*. As melanomas are felt to be “addicted” to MAPK pathway activation, identifying alternative mechanisms of MAPK pathway activation is noteworthy given its potential to contribute to our understanding of melanoma initiation. Computational analyses suggested *MAPK1*/*ERK2* as a possible target of miR-876 action, which was supported by luciferase assays showing downregulation of the *ERK* 3’ UTR and downregulation of ERK protein expression following miR-876 overexpression. Given the importance of ERK to MAPK pathway signaling, these studies suggest loss of miR-876 as an additional mechanism to activate this key pathway in melanoma. It is important to note that a prior study identified miR-524-5p [27] as targeting the *ERK* 3’ UTR in melanoma cells. However, our study also showed that *MAPK1*/*ERK2* cDNA could rescue the effects of miR-876 overexpression on melanoma colony formation, suggesting that the tumor suppressor properties of miR-876 are due, at least in part, to its targeting of ERK.

Beyond these biological implications, our study suggests the therapeutic potential of miR-876 replacement therapy in melanoma. The in vivo study using the C8161.9 model showed significant suppression of tumor growth following twice-weekly intra-tumoral injections of miR-876, underlining this therapeutic potential. While the goal of these studies was to demonstrate a functional tumor suppressor role for miR-876, it is possible that alternative treatment schedules and modes of delivery would result in a higher level of anti-tumor efficacy. In this regard, the use of lipid nanoparticles to deliver miRNAs systemically would be of interest [28]. In addition, miR-876 overexpression sensitized melanoma cells to vemurafenib treatment, suggesting a rationale for combinatorial therapy involving miR-876 overexpression and BRAF inhibition. Interestingly, a number of miRNAs have been shown to modulate sensitivity to MAPK pathway inhibitors [29], including those directly targeting MAPK pathway genes, such as miR-200c [30] and miR-579 [31].

Finally, bulk RNA-Seq analysis identified numerous differentially expressed genes following miR-876 administration, confirmed by qRT-PCR analysis of several genes with known function in tumor biology. Among the genes downregulated following miR-876 overexpression were several tumor-promoting genes, including *CDC42EP5*, *CA9*, *VEGFB, TNFRSF4* and *DDR1*. *VEGFB* is a pro-angiogenic gene that has been shown to be regulated by ERK [32], whereas *CA9* promotes tumor progression via regulation of *HIF1A* in hypoxic conditions [33]. Intriguingly, *CDC42EP5* regulates melanoma cell motility, invasion and metastasis [34] by virtue of its association with the actin cytoskeleton. Finally, *TNFRSF4* and *DDR1* are known suppressors of apoptosis [35, 36], which in the case of *TNFRSF4* occurs by virtue of upregulation of BCL2 and BCL-XL. Among the upregulated genes were *BRCA2* and *BLM*, also with demonstrated roles in suppression of tumor progression. *BRCA2* is prototypical tumor suppressor gene involved in DNA repair (through homologous recombination) and in familial cancer susceptibility [37, 38]. Finally, *BLM* encodes a DNA helicase that helps maintain the fidelity of homologous recombination [39] and is mutated in Bloom syndrome, characterized by growth retardation, immunodeficiency, and cancer susceptibility. Thus, miR-876 treatment in vivo promotes a transcriptional program that helps explain its tumor suppressor and pro-apoptotic activity.

## Conclusions

In conclusion, our studies identified miR-876 as an additional tumor suppressor on 9p21 that is inactivated in melanoma by virtue of copy number loss. In addition, they showed a functional tumor suppressor role for miR-876 in melanoma. Finally, these studies identified ERK as a downstream target of miR-876 action in melanoma, suggesting miR-876 loss as an additional mechanism to activate the MAPK pathway in melanoma, as well as indicating the potential for combining miR-876 replacement therapy with existing selective BRAF inhibitors in melanoma.

### Supplementary Information

Supplementary Material 1.

Supplementary Material 2.

Supplementary Material 3.

## Data Availability

The RNA-Seq data in this study are deposited in GEO (accession number GSE267789). All other datasets used and/or analyzed during the current study are available from the corresponding author on reasonable request.

## Abbreviations

miR-876: MicroRNA-876
qRT-PCR: Quantitative reverse transcriptase polymerase chain reaction
TCGA: The Cancer Genome Atlas
RNA-Seq: RNA sequencing
MAPK: Mitogen activated protein kinase
MTAP: Methylthiadenosine phosphorylase
IACUC: Institutional Animal Care and Use Committee
NGS: Next-generation sequencing
SNV: Single-nucleotide variant
FISH: Fluorescence in situ hybridization
BAC: Bacterial artificial chromosome clones
CEP: Chromosome enumeration probes
