# Supplementary material for: miR-876-3p is a tumor suppressor on 9p21 that is inactivated in melanoma and targets ERK
Source: J Transl Med. 2024 Aug 13;22:758. doi: 10.1186/s12967-024-05527-7 (PMC11321151; doi:10.1186/s12967-024-05527-7)
Supplement: Supplementary file 1 — Supplementary Material 1. [file 12967_2024_5527_MOESM1_ESM.docx]

Table S1. Histologic Subtypes of Nevi Assessed for miR-876 expression

Subtype Number (%)

Intradermal nevus 30 (62.5)

Compound nevus 13 (27)

Dysplastic nevus 3 (6.2%)

Junctional nevus 2 (4.1)
